# P-1548. Risk Factors for Escherichia Coli Resistance to First-line Antibiotics in Urinary Isolations in Children

**DOI:** 10.1093/ofid/ofae631.1715

**Published:** 2025-01-29

**Authors:** María Cecilia Guglielmo, Veronica Guedes

**Affiliations:** Hospital de Niños Pedro de Elizalde, bs as, Buenos Aires, Argentina; Hospital de Niños Pedro de Elizalde, bs as, Buenos Aires, Argentina

## Abstract

**Background:**

There is concern about the increase in antibiotic resistance to Sulfamethoxazole/trimethoprim (SMX/TMP) in urinary tract infections (UTI) in children. There is limited evidence on whether there are associated risk factors for this resistance. The Objective of our investigation is to evaluate if there is an association between E. Coli resistance to (SMX/TMP) and previous episodes of UTI, prophylaxis or antibiotic treatment, hospitalizations, urinary tract disorders, age and sex.

Characteristics of children with SMZ-TMP-resistant E. Coli isolates compared to the control group

**Figure:**
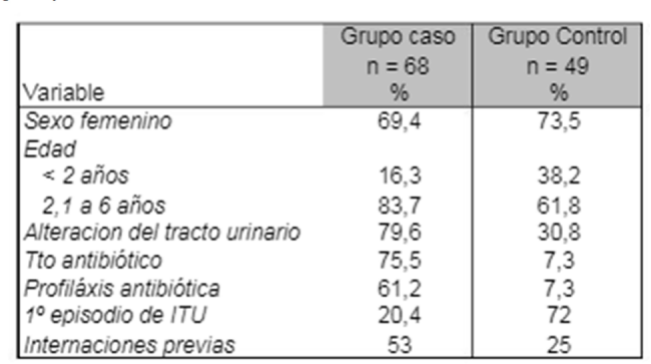

**Methods:**

retrospective study that evaluated 117 medical records from Nephrology service of Elizalde Hospital, in Buenos Aires , Argentina from 1/1/2018 to 12/31/2018. The sample was divided into two groups. A control group (68 patients) with E. coli sensitive to SMX/TMP in urine isolations and a group of cases (49 patients) with resistant isolates. Risk factors were considered and association was evaluated by Chi test, OR calculation

with 95% confidence intervals. Binary logistic regression was performed . Significance 0.05. Age was dichotomized into older and younger than 2 years old

**Results:**

Sex showed no association with resistant insulation. Patients older than 2 years old, were associated with resistant isolates (p = 0.01). Patients with hospitalizations were 3 times more likely to have a resistant insulation (p = 0.001). Alterations in the urinary tract increased 3.9 times the risk of resistant isolates (p < 0.0001). Greater resistance was observed in those with recurrence of infection (p < 0.0001). The multivariate analysis indicated that those patients with prophylaxis had an 83 times greater risk of presenting isolates resistant to SMX/TMP than patients without this risk factor (OR 83.47, 95% CI 12.6 – 552.21)

**Conclusion:**

Prolonged use of antibiotics and receiving antibiotic prophylaxis were the only

factors independently associated with the finding of SMX/TMP-resistant E. Coli

**Disclosures:**

**All Authors**: No reported disclosures

